# *N*,*N*′-(Ethane-1,2-di­yl)di­benzene­carbo­thio­amide

**DOI:** 10.1107/S1600536814008368

**Published:** 2014-04-18

**Authors:** Masayuki Nagasawa, Yuji Sasanuma, Hyuma Masu

**Affiliations:** a Department of Applied Chemistry and Biotechnology, Chiba University, 1-33 Yayoi-cho, Inage-ku, Chiba 263-8522, Japan; b Center for Analytical Instrumentation, Chiba University, 1-33 Yayoi-cho, Inage-ku, Chiba 263-8522, Japan

## Abstract

The title compound, C_16_H_16_N_2_S_2_, adopts a *gauche* ^+^–*gauche* ^+^–*gauche* ^+^ (*g^+^g^+^g^+^*) conformation in the NH—CH_2_—CH_2_—NH bond sequence. In the crystal, mol­ecules are connected by pairs of N—H⋯S=C hydrogen bonds and C—H⋯π inter­actions, forming a tape structure along the *c-*axis direction.

## Related literature   

For crystal structures and conformations of related compounds with –(C=X)–C_6_H_4_–(C=X)–Y–(CH_2_)_*m*_–Y– (X = O or S and Y = O, S, or NH) bond sequences, see for example,: Palmer & Brisse (1980 ▶); Brisson & Brisse (1986 ▶); Abe *et al.* (2011 ▶); Abe & Sasanuma (2012 ▶, 2013 ▶). For the synthesis, see: Jacobson *et al.* (1987 ▶).
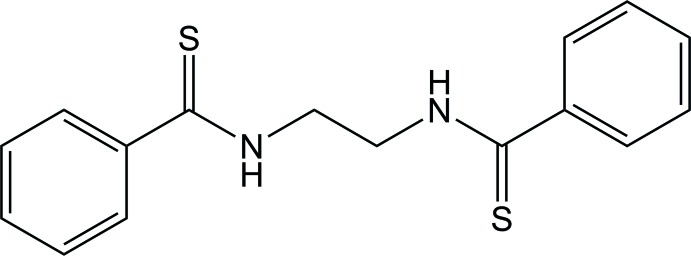


## Experimental   

### 

#### Crystal data  

C_16_H_16_N_2_S_2_
*M* *_r_* = 300.43
Triclinic, 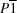

*a* = 8.6652 (1) Å
*b* = 9.4596 (1) Å
*c* = 10.3457 (1) Å
α = 105.5452 (7)°
β = 98.9293 (7)°
γ = 101.5370 (6)°
*V* = 780.67 (2) Å^3^
*Z* = 2
Cu *K*α radiation
μ = 3.01 mm^−1^
*T* = 223 K
0.20 × 0.05 × 0.05 mm

#### Data collection  

Bruker APEXII Ultra CCD area-detector diffractometer
Absorption correction: multi-scan (*SADABS*; Bruker, 2001 ▶) *T*_min_ = 0.58, *T*_max_ = 0.86
10280 measured reflections
2763 independent reflections
2568 reflections with *I* > 2σ(*I*)
*R*_int_ = 0.014

#### Refinement  

*R*[*F* ^2^ > 2σ(*F* ^2^)] = 0.029
*wR*(*F* ^2^) = 0.083
*S* = 1.04
2763 reflections
181 parameters
H-atom parameters constrained
Δρ_max_ = 0.28 e Å^−3^
Δρ_min_ = −0.18 e Å^−3^

### 

Data collection: *APEX2* (Bruker, 2007 ▶); cell refinement: *SAINT* (Bruker, 2007 ▶); data reduction: *SAINT*; program(s) used to solve structure: *SHELXS2013* (Sheldrick, 2008 ▶); program(s) used to refine structure: *SHELXL2013* (Sheldrick, 2008 ▶); molecular graphics: *Mercury* (Macrae *et al.*, 2006 ▶); software used to prepare material for publication: *SHELXL2013*.

## Supplementary Material

Crystal structure: contains datablock(s) global, I. DOI: 10.1107/S1600536814008368/is5357sup1.cif

Structure factors: contains datablock(s) I. DOI: 10.1107/S1600536814008368/is5357Isup2.hkl

Supporting information file. DOI: 10.1107/S1600536814008368/is5357Isup3.cml

CCDC reference: 997112

Additional supporting information:  crystallographic information; 3D view; checkCIF report

## Figures and Tables

**Table 1:** Hydrogen-bond geometry (Å, °) *Cg*1 and *Cg*2 are the centroids of the C2–C7 and C11–C16 phenyl rings, respectively.

*D*—H⋯*A*	*D*—H	H⋯*A*	*D*⋯*A*	*D*—H⋯*A*
N1—H1⋯S2^i^	0.87	2.56	3.4186 (13)	168
N2—H2⋯S1^ii^	0.87	2.58	3.4097 (13)	159
C8—H8*A*⋯*Cg*2^i^	0.99	2.78	3.5376 (17)	134
C9—H9*A*⋯*Cg*1^i^	0.99	2.87	3.6685 (17)	140

Symmetry codes: (i) 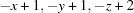; (ii) 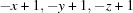.

**Fig. 1.**
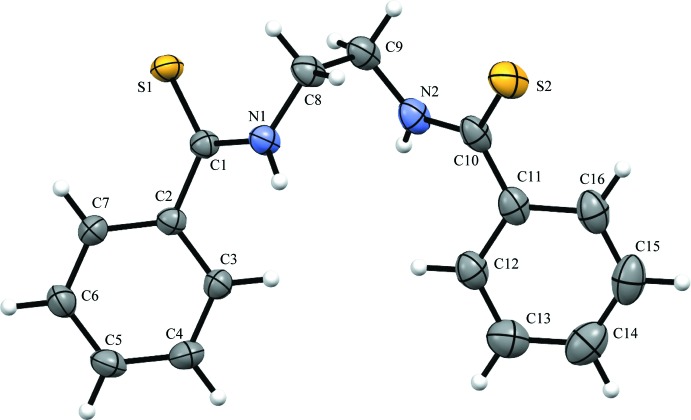
Molecular structure of the title compound. Displacement ellipsoids are drawn at the 50% probability level. Isotropic H-atom thermal parameters are represented by spheres of arbitrary size. The labels of hydrogen atoms are omitted for clarity.

**Fig. 2.**
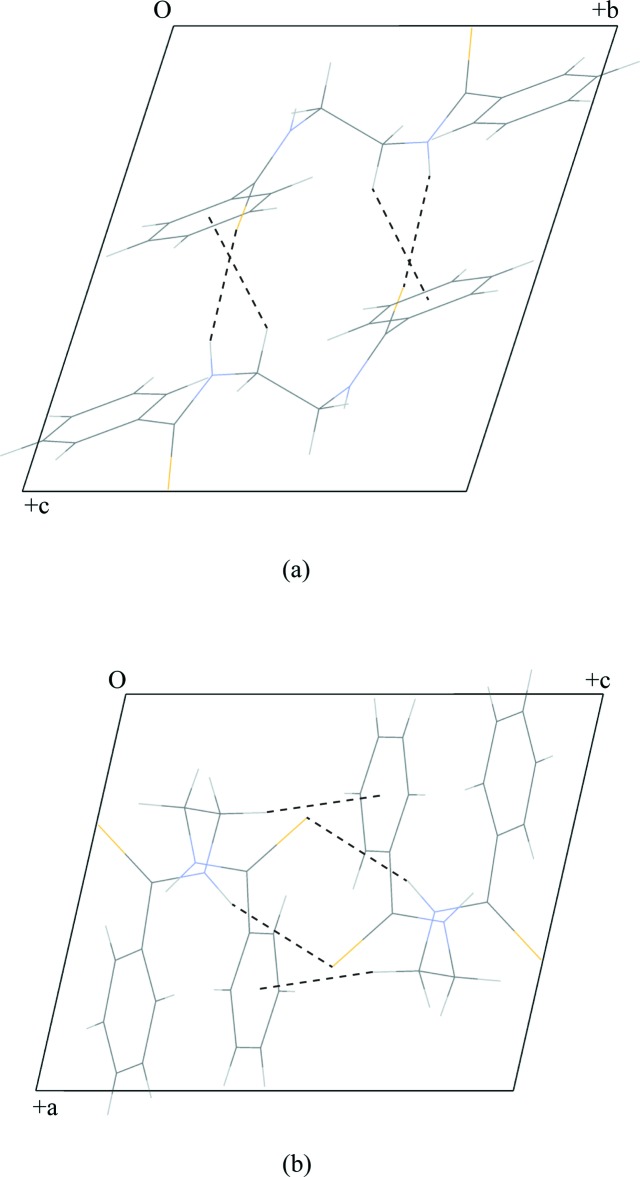
Packing diagrams of the title compound, viewed down the (a) a and (b) b axes. The dotted lines represent C=S···H—N and C—H···π interactions.

## supplementary crystallographic information

### 1. Comment

In our previous studies, conformational characteristics and configurational properties of aromatic polythioesters (X = O and Y = S) and polydithioesters (X = Y = S) (Abe & Sasanuma, 2012) expressed as [–(C=X)–C_6_H_4_–(C=X)–Y–(CH_2_)*_m_*–Y–]*_n_* were investigated through molecular orbital (MO) calculations and NMR and single-crystal X-ray diffraction experiments on their model compounds (Abe *et al.*, 2011; Abe & Sasanuma, 2013). The theoretical and experimental data thus obtained were applied to the *ab initio* statistical mechanics to derive bond conformations, configurational properties, and thermodynamic quantities on the target polymers. In the present study, we have treated aromatic polyamides (X = O and Y = NH), polythioamides (X = S and Y = NH), and their model compounds, C_6_H_5_–(C=X)–NH–(CH_2_)*_m_*–NH–(C=X)–C_6_H_5_. Crystal structures of the model compounds (X = O and *m* = 2 and 3) of poly(ethylene terephthalamide) and poly(trimethylene terephthalamide) were already determined (Palmer & Brisse, 1980; Brisson & Brisse, 1986). This paper reports the crystal structure of *N,N'*-(ethane-1,2-diyl)benzenecarbothioamide (X = S and *m* = 2, referred to hereafter as EDBTA) corresponding to the model compound of poly(ethylene terephthalthioamide).

Figure 1 shows the molecular structure of EDBTA, whose NH–CH_2_–CH_2_–NH bonds adopt the *g^+^g^+^g^+^* conformation. The MO calculations at the B3LYP/6–311+G(2d,p)//B3LYP/6–311+G(2d,p) level including the solvent effect of dimethyl sulfoxide have predicted conformational preferences of EDBTA; the first and second most stable conformers are *tg^+^g^–^* (–0.99) and *g^+^g^+^g^+^* (–0.76), respectively, where the values in the parentheses are Gibbs free energies in kcal mol^–1^ relative to that of the all-*trans* state.

According to the MO calculations, the *tg^+^g^–^* conformer of EDBTA seems to form intramolecular C=S···H–N and C=S···C–H attractions. As shown in Figure 2, the crystallized EDBTA molecule, lying in the *g^+^g^+^g^+^* conformation, forms intermolecular C=S···H–N and C–H..π interactions. Probably, the crystalline EDBTA chooses the intermolecular C=S···H–N interaction rather than the intramolecular one to acquire a larger energy stability. The MO calculations predicted that stable conformers of *N,N'*-(ethane-1,2-diyl)dibenzamide (X = O and *m* =2, abbreviated as EDBA), the model compound of poly(ethylene terephthalamide), are, in the ascending order of free energy, *tg^+^g^–^*, *g^+^tg^+^*, *g^+^g^+^g^+^*, *g^+^tg^–^*,···; the energy difference between *g^+^tg^–^* and *tg^+^g^–^* was estimated as 0.89 kcal mol^-1^. Nevertheless, the EDBA molecule crystallizes to adopt the fourth stable conformation, *g^+^tg^–^* (Palmer & Brisse, 1980). In contrast with models of the polythioester (X = O, Y = S, and *m* = 2) and polydithioester (X = Y = S and *m* = 2) (Abe *et al.*, 2011; Abe & Sasanuma, 2012), EDBA and EDBTA do not crystallize in the most stable conformation suggested by the MO calculations probably because of the significant stabilization of the intermolecular C=O···H—N and C=S···H—N hydrogen bonds.

### 2. Experimental

Benzoyl chloride (4.6 ml, 40 mmol), dissolved in 1,2-dichloroethane (100 ml), was added dropwise to ethylenediamine (14 ml, 210 mmol) and 1,2-dichloroethane (300 ml) stirred by a magnetic stirrer in a three-necked flask equipped with a dropping funnel and a calcium-chloride drying tube, with the flask being bathed in ice water. The mixture was stirred at room temperature for 8 h to yield white precipitate. The precipitate was collected by suction filtration, washed with water, and dried. The crude product was recrystallized from methanol and dried at 40 °C under reduced pressure to yield EDBA (yield 55%). In principle, this synthesis is based on the procedure of Jacobson *et al.* (1987).

Lawesson's reagent (1.8 g, 4.6 mmol) and EDBA (1.0 g, 3.7 mmol) were dissolved in toluene (20 ml) stirred in a three-necked flask equipped with a reflux condenser connected to a calcium-chloride drying tube. The solution was refluxed under dry nitrogen at *ca* 110 °C for 8 h to yield yellow precipitate. The precipitate was collected, washed with toluene, recrystallized from ethanol, and dried at 40 °C under reduced pressure to yield EDBTA (yield 79%).

A small quantity of EDBTA was dissolved in chloroform in a glass tube, whose top was sealed with a thin Teflon film. The tube was placed in a vial container including a small amount of *n*-hexane, and the container was capped and left to stand still in a dark place. After a day, its crystals were found to be formed in the inner tube.

### 3. Refinement

All H atoms were geometrically positioned with C—H = 0.95 and 0.99 Å for the aromatic and methylene groups, respectively, and N—H = 0.87 Å, and refined as riding by *U*_iso_(H) = 1.2*U*_eq_(C, N).

### Crystal data

**Table:**

C_16_H_16_N_2_S_2_	*V* = 780.67 (2) Å^3^
*M**_r_* = 300.43	*Z* = 2
Triclinic, *P*1	*F*(000) = 316
*a* = 8.6652 (1) Å	*D*_x_ = 1.278 Mg m^−^^3^
*b* = 9.4596 (1) Å	Cu *K*α radiation, λ = 1.54178 Å
*c* = 10.3457 (1) Å	µ = 3.01 mm^−^^1^
α = 105.5452 (7)°	*T* = 223 K
β = 98.9293 (7)°	Needle, yellow
γ = 101.5370 (6)°	0.20 × 0.05 × 0.05 mm

### Data collection

**Table:**

Bruker APEXII Ultra CCD area-detector diffractometer	2763 independent reflections
Radiation source: Bruker TXS fine-focus rotating anode	2568 reflections with *I* > 2σ(*I*)
Bruker Helios multilayer mirror monochromator	*R*_int_ = 0.014
φ and ω scans	θ_max_ = 68.2°, θ_min_ = 4.6°
Absorption correction: multi-scan (*SADABS*; Bruker, 2001)	*h* = −10→10
*T*_min_ = 0.58, *T*_max_ = 0.86	*k* = −11→11
10280 measured reflections	*l* = −10→12

### Refinement

**Table:**

Refinement on *F*^2^	0 restraints
Least-squares matrix: full	Hydrogen site location: inferred from neighbouring sites
*R*[*F*^2^ > 2σ(*F*^2^)] = 0.029	H-atom parameters constrained
*wR*(*F*^2^) = 0.083	*w* = 1/[σ^2^(*F*_o_^2^) + (0.0481*P*)^2^ + 0.2337*P*] where *P* = (*F*_o_^2^ + 2*F*_c_^2^)/3
*S* = 1.04	(Δ/σ)_max_ = 0.001
2763 reflections	Δρ_max_ = 0.28 e Å^−^^3^
181 parameters	Δρ_min_ = −0.18 e Å^−^^3^

### Special details

**Table:**

Geometry. All e.s.d.'s (except the e.s.d. in the dihedral angle between two l.s. planes) are estimated using the full covariance matrix. The cell e.s.d.'s are taken into account individually in the estimation of e.s.d.'s in distances, angles and torsion angles; correlations between e.s.d.'s in cell parameters are only used when they are defined by crystal symmetry. An approximate (isotropic) treatment of cell e.s.d.'s is used for estimating e.s.d.'s involving l.s. planes.
Refinement. Refinement of *F*^2^ was performed with all reflections. The weighted *R*-factor (*wR*) and goodness of fit (*S*) are based on *F*^2^, while the *R*-factor on *F*. The threshold expression of *F*^2^ > 2.0 σ(*F*^2^) was used only for calculating *R*-factor.

### Fractional atomic coordinates and isotropic or equivalent isotropic displacement parameters (Å^2^)

**Table:**

	*x*	*y*	*z*	*U*_iso_*/*U*_eq_	
C1	0.55284 (15)	0.70259 (14)	0.66245 (14)	0.0269 (3)	
C2	0.39798 (16)	0.74362 (15)	0.62755 (14)	0.0267 (3)	
C3	0.25373 (17)	0.65794 (16)	0.63997 (14)	0.0306 (3)	
H3	0.2543	0.5762	0.6752	0.037*	
C4	0.10937 (17)	0.69267 (17)	0.60064 (16)	0.0356 (3)	
H4	0.0121	0.6341	0.6086	0.043*	
C5	0.10820 (18)	0.81363 (18)	0.54961 (16)	0.0373 (3)	
H5	0.0101	0.8369	0.5226	0.045*	
C6	0.25101 (18)	0.90021 (18)	0.53830 (17)	0.0379 (3)	
H6	0.2501	0.9829	0.5044	0.046*	
C7	0.39529 (17)	0.86538 (16)	0.57682 (15)	0.0325 (3)	
H7	0.4922	0.9244	0.5687	0.039*	
C8	0.71343 (18)	0.60757 (18)	0.82305 (15)	0.0353 (3)	
H8A	0.7237	0.6194	0.9213	0.042*	
H8B	0.8119	0.6707	0.8121	0.042*	
C9	0.69808 (19)	0.44220 (18)	0.74548 (16)	0.0374 (3)	
H9A	0.7012	0.4325	0.6493	0.045*	
H9B	0.7903	0.4104	0.7855	0.045*	
C10	0.52445 (19)	0.29310 (17)	0.85582 (15)	0.0360 (3)	
C11	0.35708 (19)	0.20806 (17)	0.84557 (15)	0.0363 (3)	
C12	0.2252 (2)	0.25356 (18)	0.79138 (16)	0.0400 (4)	
H12	0.2417	0.3354	0.7556	0.048*	
C13	0.0696 (2)	0.1792 (2)	0.78978 (18)	0.0504 (4)	
H13	−0.0187	0.2121	0.7548	0.06*	
C14	0.0438 (2)	0.0564 (2)	0.8396 (2)	0.0569 (5)	
H14	−0.0618	0.0058	0.8383	0.068*	
C15	0.1734 (3)	0.0089 (2)	0.89112 (19)	0.0553 (5)	
H15	0.1559	−0.0756	0.9235	0.066*	
C16	0.3285 (2)	0.08408 (19)	0.89556 (17)	0.0453 (4)	
H16	0.4162	0.0517	0.9326	0.054*	
N1	0.57488 (14)	0.66031 (14)	0.77434 (12)	0.0312 (3)	
H1	0.5012	0.6642	0.8226	0.037*	
N2	0.54942 (16)	0.34276 (14)	0.75044 (12)	0.0361 (3)	
H2	0.4702	0.3133	0.6785	0.043*	
S1	0.68758 (4)	0.70858 (4)	0.56244 (4)	0.03447 (12)	
S2	0.67017 (5)	0.32702 (6)	0.99595 (4)	0.04788 (14)	

### Atomic displacement parameters (Å^2^)

**Table:**

	*U*^11^	*U*^22^	*U*^33^	*U*^12^	*U*^13^	*U*^23^
C1	0.0259 (6)	0.0285 (6)	0.0259 (7)	0.0067 (5)	0.0055 (5)	0.0083 (5)
C2	0.0270 (6)	0.0306 (6)	0.0236 (7)	0.0092 (5)	0.0072 (5)	0.0080 (5)
C3	0.0319 (7)	0.0325 (7)	0.0311 (8)	0.0095 (6)	0.0100 (6)	0.0130 (6)
C4	0.0262 (7)	0.0414 (8)	0.0396 (9)	0.0069 (6)	0.0104 (6)	0.0125 (6)
C5	0.0299 (7)	0.0492 (9)	0.0379 (9)	0.0186 (6)	0.0074 (6)	0.0154 (7)
C6	0.0407 (8)	0.0415 (8)	0.0422 (9)	0.0190 (7)	0.0127 (7)	0.0220 (7)
C7	0.0303 (7)	0.0356 (7)	0.0366 (8)	0.0092 (6)	0.0116 (6)	0.0164 (6)
C8	0.0350 (7)	0.0483 (8)	0.0265 (8)	0.0169 (6)	0.0040 (6)	0.0146 (6)
C9	0.0429 (8)	0.0509 (9)	0.0288 (8)	0.0240 (7)	0.0129 (6)	0.0176 (6)
C10	0.0488 (9)	0.0408 (8)	0.0268 (8)	0.0243 (7)	0.0112 (6)	0.0129 (6)
C11	0.0507 (9)	0.0385 (8)	0.0238 (8)	0.0186 (7)	0.0097 (6)	0.0102 (6)
C12	0.0493 (9)	0.0439 (8)	0.0310 (8)	0.0175 (7)	0.0090 (7)	0.0142 (6)
C13	0.0482 (10)	0.0628 (11)	0.0392 (10)	0.0172 (8)	0.0077 (7)	0.0128 (8)
C14	0.0600 (11)	0.0595 (11)	0.0434 (11)	0.0015 (9)	0.0154 (8)	0.0110 (8)
C15	0.0809 (14)	0.0453 (9)	0.0417 (10)	0.0100 (9)	0.0196 (9)	0.0175 (8)
C16	0.0657 (11)	0.0440 (9)	0.0324 (9)	0.0218 (8)	0.0114 (7)	0.0158 (7)
N1	0.0325 (6)	0.0418 (6)	0.0256 (6)	0.0160 (5)	0.0096 (5)	0.0140 (5)
N2	0.0444 (7)	0.0439 (7)	0.0251 (6)	0.0188 (6)	0.0073 (5)	0.0138 (5)
S1	0.02655 (19)	0.0504 (2)	0.0359 (2)	0.01414 (15)	0.01281 (14)	0.02216 (16)
S2	0.0469 (2)	0.0748 (3)	0.0317 (2)	0.0247 (2)	0.00711 (17)	0.0264 (2)

### Geometric parameters (Å, º)

**Table:**

C1—N1	1.3216 (17)	C9—H9A	0.98
C1—C2	1.4882 (18)	C9—H9B	0.98
C1—S1	1.6783 (13)	C10—N2	1.3278 (19)
C2—C7	1.3906 (19)	C10—C11	1.484 (2)
C2—C3	1.3915 (19)	C10—S2	1.6791 (15)
C3—C4	1.384 (2)	C11—C12	1.390 (2)
C3—H3	0.94	C11—C16	1.397 (2)
C4—C5	1.384 (2)	C12—C13	1.386 (2)
C4—H4	0.94	C12—H12	0.94
C5—C6	1.381 (2)	C13—C14	1.385 (3)
C5—H5	0.94	C13—H13	0.94
C6—C7	1.382 (2)	C14—C15	1.376 (3)
C6—H6	0.94	C14—H14	0.94
C7—H7	0.94	C15—C16	1.377 (3)
C8—N1	1.4598 (17)	C15—H15	0.94
C8—C9	1.523 (2)	C16—H16	0.94
C8—H8A	0.98	N1—H1	0.87
C8—H8B	0.98	N2—H2	0.87
C9—N2	1.452 (2)		
			
N1—C1—C2	115.89 (11)	N2—C9—H9B	109.2
N1—C1—S1	123.18 (10)	C8—C9—H9B	109.2
C2—C1—S1	120.93 (10)	H9A—C9—H9B	107.9
C7—C2—C3	119.15 (12)	N2—C10—C11	116.16 (13)
C7—C2—C1	119.98 (12)	N2—C10—S2	122.96 (13)
C3—C2—C1	120.82 (12)	C11—C10—S2	120.86 (11)
C4—C3—C2	120.26 (13)	C12—C11—C16	118.40 (16)
C4—C3—H3	119.9	C12—C11—C10	120.86 (13)
C2—C3—H3	119.9	C16—C11—C10	120.69 (14)
C5—C4—C3	120.02 (13)	C13—C12—C11	120.52 (15)
C5—C4—H4	120.0	C13—C12—H12	119.7
C3—C4—H4	120.0	C11—C12—H12	119.7
C6—C5—C4	120.08 (13)	C14—C13—C12	120.19 (17)
C6—C5—H5	120.0	C14—C13—H13	119.9
C4—C5—H5	120.0	C12—C13—H13	119.9
C5—C6—C7	120.05 (13)	C15—C14—C13	119.69 (18)
C5—C6—H6	120.0	C15—C14—H14	120.2
C7—C6—H6	120.0	C13—C14—H14	120.2
C6—C7—C2	120.42 (13)	C14—C15—C16	120.44 (17)
C6—C7—H7	119.8	C14—C15—H15	119.8
C2—C7—H7	119.8	C16—C15—H15	119.8
N1—C8—C9	112.27 (12)	C15—C16—C11	120.74 (16)
N1—C8—H8A	109.1	C15—C16—H16	119.6
C9—C8—H8A	109.1	C11—C16—H16	119.6
N1—C8—H8B	109.1	C1—N1—C8	124.96 (12)
C9—C8—H8B	109.1	C1—N1—H1	117.5
H8A—C8—H8B	107.9	C8—N1—H1	117.5
N2—C9—C8	112.14 (12)	C10—N2—C9	125.36 (13)
N2—C9—H9A	109.2	C10—N2—H2	117.3
C8—C9—H9A	109.2	C9—N2—H2	117.3
			
N1—C1—C2—C7	140.90 (14)	S2—C10—C11—C16	−38.65 (19)
S1—C1—C2—C7	−39.89 (17)	C16—C11—C12—C13	1.3 (2)
N1—C1—C2—C3	−41.60 (18)	C10—C11—C12—C13	−176.08 (15)
S1—C1—C2—C3	137.61 (12)	C11—C12—C13—C14	−1.4 (3)
C7—C2—C3—C4	0.8 (2)	C12—C13—C14—C15	0.2 (3)
C1—C2—C3—C4	−176.74 (13)	C13—C14—C15—C16	1.1 (3)
C2—C3—C4—C5	−0.4 (2)	C14—C15—C16—C11	−1.2 (3)
C3—C4—C5—C6	−0.2 (2)	C12—C11—C16—C15	0.0 (2)
C4—C5—C6—C7	0.6 (2)	C10—C11—C16—C15	177.37 (15)
C5—C6—C7—C2	−0.2 (2)	C2—C1—N1—C8	176.47 (13)
C3—C2—C7—C6	−0.5 (2)	S1—C1—N1—C8	−2.7 (2)
C1—C2—C7—C6	177.09 (13)	C9—C8—N1—C1	−79.82 (18)
N1—C8—C9—N2	−55.40 (17)	C11—C10—N2—C9	172.70 (13)
N2—C10—C11—C12	−39.7 (2)	S2—C10—N2—C9	−5.6 (2)
S2—C10—C11—C12	138.62 (13)	C8—C9—N2—C10	−79.40 (17)
N2—C10—C11—C16	143.00 (15)		

### Hydrogen-bond geometry (Å, º)

*Cg*1 and *Cg*2 are the centroids of the C2–C7 and C11–C16 phenyl rings, respectively.

**Table:**

*D*—H···*A*	*D*—H	H···*A*	*D*···*A*	*D*—H···*A*
N1—H1···S2^i^	0.87	2.56	3.4186 (13)	168
N2—H2···S1^ii^	0.87	2.58	3.4097 (13)	159
C8—H8*A*···*Cg*2^i^	0.99	2.78	3.5376 (17)	134
C9—H9*A*···*Cg*1^i^	0.99	2.87	3.6685 (17)	140

Symmetry codes: (i) −*x*+1, −*y*+1, −*z*+2; (ii) −*x*+1, −*y*+1, −*z*+1.
